# In this Issue

**Published:** 2009

## A DEVELOPMENTAL PERSPECTIVE ON UNDERAGE ALCOHOL USE

Alcohol use is a pervasive problem among adolescents in the United States. Childhood and adolescence are characterized by multiple developmental changes in all domains of life. Therefore, addressing underage alcohol use as a developmental phenomenon and understanding how it is shaped by the course and context of human development might be a promising approach to preventing and reducing underage drinking and its adverse effects, write Drs. Ann S. Masten, Vivian B. Faden, Robert A. Zucker, and Linda P. Spear. To support this view, the authors discuss the age-related patterns of alcohol use, abuse, and dependence as well as their consequences; the effects of alcohol on development; childhood factors that might predict later alcohol and other drug use; and age-related risk and protective factors associated with alcohol use and dependence. (pp. 3–15)

## DEVELOPMENTAL PROCESSES AND MECHANISMS: AGES 0–10

Although children in the age-group 0–10 years have a relatively low rate of alcohol use, numerous nonspecific and specific risk factors for subsequent alcohol use already are evident in this age-group. According to Drs. Robert A. Zucker, John E. Donovan, Ann S. Masten, Margaret E. Mattson, and Howard B. Moss, nonspecific risk factors that increase the risk not only of alcohol use and abuse but also of other drug use and behavioral problems include externalizing and internalizing behaviors, as well as environmental and social factors (e.g., stress, physical abuse, or other aspects of social interaction). In addition, certain nonspecific childhood factors (i.e., predictors) of childhood and early adolescent drinking have been identified that can be used to target specific population subgroups for preventive interventions. These nonspecific influences interact with alcohol-specific childhood risk factors for future alcohol use, such as age of initiation of drinking and parental drinking or a family history of alcohol dependence. The interplay of these diverse nonspecific and specific biological, psychological, and social processes shape children’s risk of alcohol use and related problems as they enter puberty. (pp. 16–29)

## TRANSITIONS INTO UNDERAGE AND PROBLEM DRINKING: SUMMARY OF DEVELOPMENTAL PROCESSES AND MECHANISMS: AGES 10–15

Dramatic changes in biological, cognitive, emotional, and social development as well as in physical and social environments are the hallmark of early adolescence (i.e., ages 10–15). Moreover, it is during this period that many adolescents begin to use alcohol. Drs. Michael Windle, Linda P. Spear, Andrew J. Fuligni, Adrian Angold, Jane D. Brown, Daniel Pine, Greg T. Smith, Jay Giedd, and Ronald E. Dahl review some of the major developmental processes and mechanisms that occur in this age-group which are related to alcohol use. These include the physiological changes associated with puberty and with continuing brain development. Equally important, family, peer, and romantic relationships change and evolve during early adolescence. The authors discuss the role of these influences, as well as their interaction with alcohol-specific and nonspecific risk factors for adolescent alcohol use in shaping adolescents’ expectancies regarding alcohol consumption and its effects. (pp. 30–40)

## UNDERAGE ALCOHOL USE: SUMMARY OF DEVELOPMENTAL PROCESSES AND MECHANISMS: AGES 16–20

Late adolescence is characterized by significant changes in neurological and cognitive processes, behavioral and social functioning, relationships, and physical contexts as the individual moves toward adulthood, and major role transitions affect almost every aspect of life. These changes impact drinking behavior during late adolescence, and alcohol use and binge drinking are particularly prevalent during this period. In this article, Drs. Sandra A. Brown, Matthew McGue, Jennifer Maggs, John Schulenberg, Ralph Hingson, Scott Swartzwelder, Christopher Martin, Tammy Chung, Susan F. Tapert, Kenneth Sher, Ken C. Winters, Cherry Lowman, and Stacia Murphy review some of the major developmental processes, transitions, and tasks in late adolescence as they relate to alcohol use and its consequences, including developmentally related effects and alcohol-specific risk and protective factors. They also discuss the alcohol use patterns of this age-group and the consequences of adolescent alcohol use and abuse. (pp. 41–52)

## OVERVIEW OF PREVENTIVE INTERVENTIONS ADDRESSING UNDERAGE DRINKING: STATE OF THE EVIDENCE AND STEPS TOWARD PUBLIC HEALTH IMPACT

Underage drinking, which is associated with numerous potential adverse consequences, is a serious public health concern. However, although many interventions aimed at adolescents of various age-groups have been developed, their effectiveness rarely has been assessed rigorously. This article by Drs. Richard Spoth, Mark Greenberg, and Robert Turrisi summarizes the results of a recent literature review to determine the evidence for the effectiveness of various existing interventions. They also highlight areas in intervention research that could be strengthened. These include increased coverage of understudied areas of intervention (e.g., specific types of interventions or interventions in specific populations), a greater focus on longitudinal studies that also provide information on alcohol-specific outcomes, as well as the availability of replication studies. Moreover, greater consistency in the standards for determining and reporting evidence of effectiveness in different studies is needed. Finally, adoption of public health impact–oriented models to accurately determine the potential of existing interventions to prevent underage drinking and its consequences can improve the relevance and reliability of prevention research in the area of underage drinking. (pp. 53–66)

## IMPROVING TREATMENT THROUGH RESEARCH: DIRECTING ATTENTION TO THE ROLE OF DEVELOPMENT IN ADOLESCENT TREATMENT SUCCESS

Although treatment of alcohol problems in adolescents can be effective in the short term, many patients resume drinking within a few months. According to Dr. Eric F. Wagner, one factor that may contribute to the high relapse rates is the substantial variation among adolescents in developmental stage. However, few studies to date have assessed the association between developmental stage and treatment outcome. As Dr. Wagner explains, greater consensus is needed on which outcomes should be evaluated in adolescent treatment studies before meaningful studies can be designed. In addition, concepts of developmental science should be integrated into adolescent treatment research to determine which factors and influences impact treatment outcome. Only with this additional knowledge can researchers and clinicians design and implement more effective treatment approaches for adolescents. (pp. 67–75)

## CURRENT STATE OF TREATMENT FOR ALCOHOL AND OTHER DRUG USE DISORDERS IN ADOLESCENTS

Researchers and clinicians now are recognizing that the treatment needs of adolescents with alcohol problems differ from those of adults. Thus, adolescents have specific developmental characteristics that may influence treatment design, patient adherence to treatment, and treatment outcome. As a result of this realization, some treatment approaches have been developed to specifically target adolescents with alcohol and other drug use problems. Most of these involve psychosocial approaches, such as family-based interventions, motivational enhancement therapy, behavioral therapy, and cognitive–behavioral therapy. As Drs. Deborah Deas and Andrew Clark report, a range of outcome studies have evaluated the success of the various approaches, and this article summarizes the findings from and limitations of these outcome studies. The authors conclude that although existing research indicates that different strategies can improve the adolescents’ outcomes on a variety of measures, use of more consistent, state-of-the-art assessment instruments and greater attention to the adolescents’ developmental status can further enhance treatment success. (pp. 76–82)

*This issue of* Alcohol Research & Health *spotlights findings from articles that originally appeared in the American Academy of Pediatrics journal Pediatrics (Vol. 121, Supplement, 2008).*

